# Distribution and species diversity of the floating green macroalgae and micro-propagules in the Subei Shoal, southwestern Yellow Sea

**DOI:** 10.7717/peerj.10538

**Published:** 2020-12-17

**Authors:** Xiaoxiang Miao, Jie Xiao, Qinzeng Xu, Shiliang Fan, Zongling Wang, Xiao Wang, Xuelei Zhang

**Affiliations:** 1 College of Environmental Science and Engineering, Ocean University of China, Qingdao, China; 2 Key Laboratory of Marine Eco-Environmental Science and Technology, First Institute of Oceanography, Ministry of Natural Resources, Qingdao, China; 3 Laboratory of Marine Ecology and Environmental Science, Pilot National Laboratory for Marine Science and Technology (Qingdao), Qingdao, China

**Keywords:** Green tides, Micro-propagules, Macroalgae, *Ulva prolifera*, Yellow Sea

## Abstract

Massive floating green macroalgae have formed harmful green tides in the Yellow Sea since 2007. To study the early development and the associated environmental factors for the green tide, a field survey was carried out in the Subei Shoal, southwestern Yellow Sea. Multiple species were identified in both floating green macroalgae and micro-propagules , while their abundances showed distinct spatial variations. The floating macroalgal biomass was widespread in the northern Subei Shoal and most abundant at 34°N. *Ulva prolifera* dominated (91.2% in average) the floating macroalgae, and the majority (88.5%) of *U. prolifera* was the ‘floating type’. In comparison, the micro-propagules were most abundant around the aquaculture rafts, and decreased significantly with the distance to the rafts. The dominant species of micro-propagules was *U. linza* (48.5%), followed by *U. prolifera* (35.1%). Their distinct distribution patterns and species diversity suggested little direct contribution of micro-propagules for the floating macroalgae. The spatial variation of the floating macroalgae was probably a combined result from the biomass source and environmental factors, while the abundance of micro-propagules was closely associated with the rafts. A positive correlation between the floating macroalgae and DO was observed and suggested active photosynthesis of the initial biomass in Subei Shoal. This study revealed specific distributional pattern and relationships among the floating macroalgae, micro-propagules and the environmental factors in the source region, which helps understanding the early blooming dynamics of the green tides in Yellow Sea.

## Introduction

In June of 2008, massive floating green macroalgae biomass aggregated in the coastal area of Qingdao, covering the sea surface of approximately 400 km^2^, formed an astonishing green tide and threatened the Olympic Sailing Regatta (BCMES, 2008; Hu, 2009; Leliaert et al., 2009; Ye et al., 2011). Thereafter, the large-scale green tides recurred annually in southern Yellow Sea, becoming an ecological disaster due to its high biomass, large distributional range and significant economic losses and social impacts (Liu et al., 2009; Liu et al., 2013; Hu et al., 2010; Ye et al., 2011). The major blooming species was identified to be *Ulva prolifera* (Leliaert et al., 2009; Wang et al., 2010; Hiraoka et al., 2011). Recently, a ‘floating’ ecotype was found widespread in the floating algae and considered to be the dominant genotype of the green tides in Yellow Sea (Zhao et al., 2015; Jiang & Zhao, 2018; Zhang et al., 2018).

The general raft-origin and northward drifting process of the Yellow Sea green tides (YSGT) were revealed and confirmed by various studies. Originally, satellite remote sensing analyses indicated that the green tide was initiated from the southwestern Yellow Sea, close to Subei Shoal (Liu et al., 2009; Hu et al., 2010). Further field surveys found that the green macroalgal wastes from the aquaculture rafts contributed substantial initial biomass for the floating algae (Liu et al., 2009; Liu et al., 2012; Liu et al., 2013; Wang et al., 2015; Zhou et al., 2015). Subsequent geophysical experiments and numeric modeling suggested that the initial floating algae then drifted out of Subei Shoal and spread throughout the open waters in the north and the offshore regions of Yellow Sea driven by the monsoon wind and strong tidal currents (Keesing et al., 2011; Bao et al., 2015; Zhang et al., 2017; Wang et al., 2018). The other studies revealed series of physiological features of *U. prolifera* and environmental parameters in southern Yellow Sea, which assisted and favored blooming of this species (Li et al., 2015; Shi et al., 2015; Xiao et al., 2016).

Micro-propagules are gametes, spores, zygotes, micro-germlings and vegetative fragments of macroalgae (Hoffmann & Santelices, 1991; Liu et al., 2012). Initial laboratory trials suggested that micro-propagules of the green seaweeds had a strong resistance to the harsh environment (such as darkness, low temperature and irradiation) and hypothesized that they could serve as the ‘seeds’ for the future blooms in suitable conditions (Lotze et al., 1999). A number of field observations in Yellow Sea detected evident temporal and spatial variations of the green macroalgal micro-propagules in large-scale offshore water, which was likely related to the dispersed floating macroalgae (Huo et al., 2014; Huo et al., 2016; Li et al., 2014; Song et al., 2014; Wang et al., 2018). The following study revealed distinct species succession of the micro-propagule communities in the near-shore coasts, indicating probably different population dynamics and function of the micro-propagule communities in different regions (Miao et al., 2018; Miao et al., 2020).

Little, however, was known about the detailed species composition and succession of the micro-propagules and any relationship with the attached and floating algae in a micro-geographicscale, especially in Subei Shoal, where the green tide was initiated and both raft-attached and free-floating macroalgae existed. The source of the micro-propagules and their function in green tide remained unclear so far. As described above, abundance of micro-propagules likely followed the spreading of the floating *Ulva* algae in open waters (Li et al., 2014), while that in inshore water showed seasonal fluctuation (Miao et al., 2018; Miao et al., 2020). Previous research revealed an evident spatial gradient of the micro-propagules in Subei Shoal (Fang et al., 2012; Song et al., 2015), which indicated the suitable environmental conditions of the Subei Shoal for maintaining abundant micro-propagules, including high ratio of *U. prolfiera* (Miao et al., 2020). Then, further questions are brought out on what kind of environmental factors of the shoal could control or even regulate the micro-propagules, and how these periodic biological processes in the shoal correlated together to drive the successive green tides. In this study, a field cruise was conducted in and around Subei Shoal in May of 2016 to survey the abundance and community structure of the floating algae and micro-propagules, and the environmental parameters. Correlations were further analyzed to detect any associations among the floating algae, micro-propagules and the various environmental factors, which would provide fundamental data for YSGT forecasting and prevention at the early stage.

## Materials and Methods

### Sampling area and stations

The survey was conducted in the coastal water of Subei Shoal in southern Yellow Sea (32.33°∼34.00°N, 120.50°∼122.17°E). Subei Shoal is located off the coast of southern Jiangsu Province, and enclosed over 70 sand ridges extending radically from central region to the offshore. With the strong tidal force, the shallow water in this region was highly turbid and well mixed. Given the shallow and nutrient-rich water column (Zhou et al., 2015), *Pyropia yezoensis* aquaculture has been conducting since 1980s and becomes the most important industry in this region (Liu et al., 2017; Wei et al., 2018).

**Figure 1:**
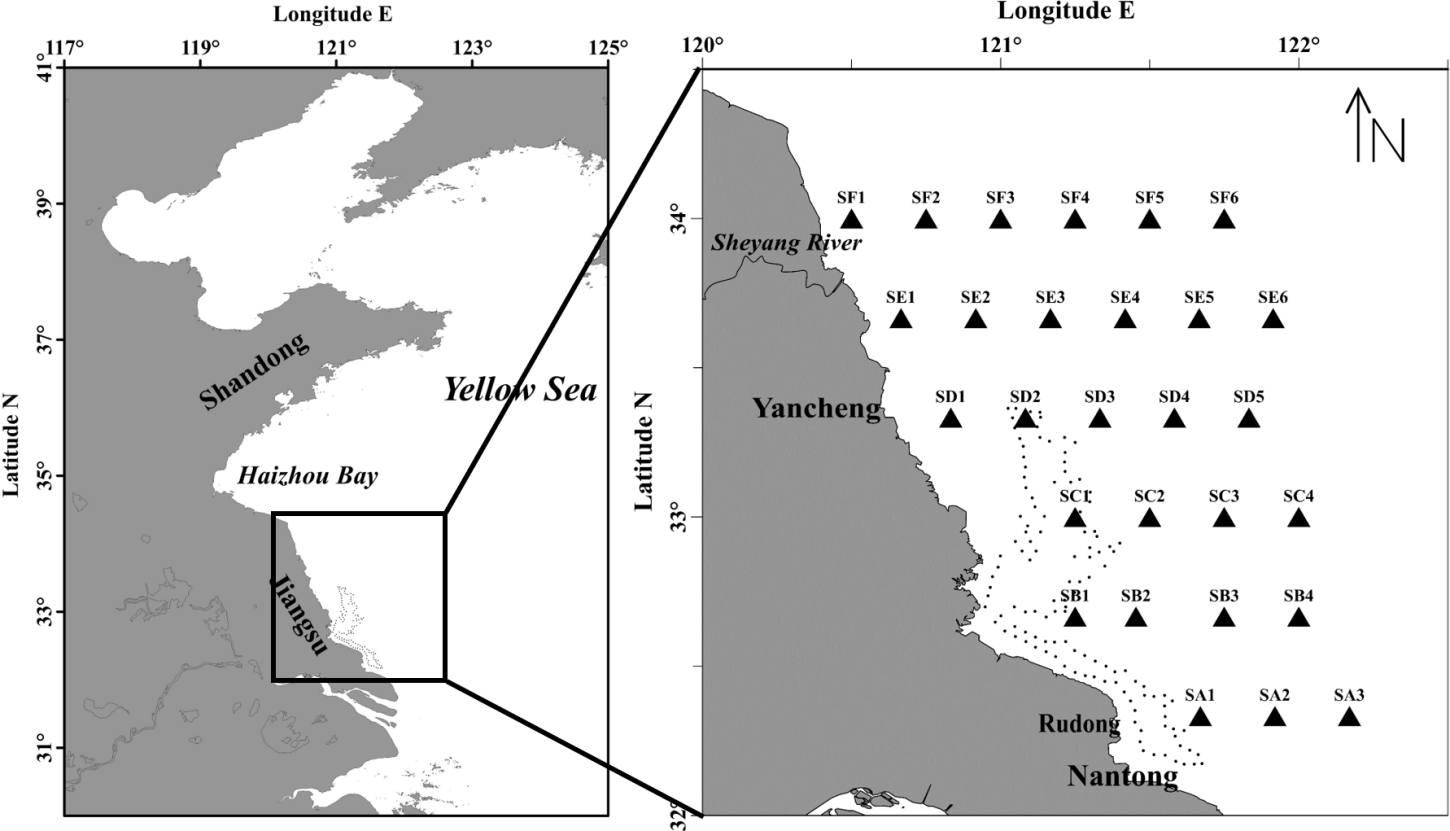
Maps of the study region (A) and sampling stations (B) in the southwestern Yellow Sea. The dotted line encloses the distribution of *Pyropia* aquaculture rafts.

The cruise was carried out during May 7th–20th 2016, when the green tide was initiated in Subei Shoal. The research vessel navigated along 6 transects (SA, SB, SC, SD, SE and SF) and samples were collected at 28 stations to investigate the distribution, abundance, species composition of the floating algae and micro-propagules, and the environmental factors as well (Fig. 1).

### Bio-trawling the biomass of floating algae

The biomass of the floating macroalgae was collected through a WP2 zooplankton net with the mesh size 500 µm and inner opening diameter 0.8 m. The net was mounted on the side of the vessel, towed at 3 knots for 10 min to collect the floating green macroalgae. Green macroalgal samples were then rinsed with filtered seawater, drained until no dripping water and then weighted using an electronic balance for wet weight.

The average biomass $\bar {W}$ (g m^−2^) was calculated as follows: $\bar {W}$ =W∕(d × L), where W is the fresh weight of floating green algae (g); d is the inner opening diameter of the WP2 zooplankton net (m); L is the trawling distance (m).

After weighing, the green macroalgae were randomly sampled and frozen in eppendorf tubes for following molecular identification. For the station with green macroalgal biomass <1 g m^−2^, 5 samples were collected for species identification. For the station with biomass ranged between 1–20 g m^−2^, 10 samples were sampled. For the others (>20 g m^−2^ in each station), we randomly selected 20 individual thalli for identification.

### Culturing micro-propagules

As described above, the seawater in Subei Shoal was well mixed. Therefore the surface seawater was collected by a Water-sampler (HQM-1, Juchuang Inc., Qingdao, CHN) at each station. The seawater sample was stored in a plastic carboy after filtering through a 200 µm mesh net to remove the major zooplanktons. The samples were stored at 4 °C until they were transported to the laboratory for culturing.

In the laboratory, one liter of seawater was cultured in a glass beaker with 20 mL Provasoli-enriched seawater medium (PES, Berges, Franklin & Harrison, 2001) and saturated GeO_2_ (final concentration 0.5 mg mL^−1^) to inhibit the growth of diatoms (Liu et al., 2012). Triplicate treatments were set up for each water sample in an Artificial Climatic Chamber (202728-380, Jiangnan Inc., Ningbo, China) at 16 °C with 100 µmol photons m^−2^s^−1^ and 12 h: 12 h light: dark light cycle. The medium was renewed every 5 days to maintain sufficient nutrients for the growth of micro-propagules. After about 20 days, the micro-propagules developed into visible germlings. The number of germlings was counted and considered as the total number of micro-propagules in the water sample. The abundance of micro-propagules (A, inds L^−1^) was then calculated as A = N∕V (N: total number of germlings, V: volume of water sample cultured).

### Species identification of floating green macroalgae and micro-propagules

The green macroalgal samples and germlings derived from micro-propagules were rinsed with deionized water for three times to remove the debris and sediments. DNA was extracted using the Chelex 100 (Miao et al., 2018). Species identification of the green algae was conducted following the protocol of Xiao et al. (2013). In brief, *U. prolifera* / *U. linza* was differentiated from the other *Ulva* common species based on PCR-RFLP of the ITS sequences. As was shown by Xiao et al. (2013), *U. prolifera* and *U. linza* differ from other *Ulva* spp. by allele sizes of restriction fragment of ITS. Then, *U. linza* and *U. prolifera* was further distinguished by PCR amplification of the 5S spacer fragments (Yotsukura et al., 2002; Shimada et al., 2003). To tracing the unique ‘floating’ ecotype type of *U. prolifera* dominating in the large-scale green tide in Yellow Sea, a third-round PCR amplification of SCAR (sequence characterized amplified region) was conducted for the specimen identified as *U. prolifera*. The SCAR marker was amplified using YSF-F (forward) and YSF-R (reverse) primers (Zhao et al., 2015). PCR products were examined on 1% agarose gel, and the ‘floating type’ was determined based on the presence of a specific 830 bp band.

### Temperature, salinity, DO, Chl a and nutrients of the surface seawater

Surface seawater temperature (SST) and salinity were measured and recorded in situ using the Multi-Parameter Water Quality Detector (YSI, USA). Dissolved oxygen was measured on board by Winkler titration, as described in Grasshoff, Kremling & Ehrhardt (1999). To measure the Chl *a* and nutrients, seawater was filtered through the 0.45 µm Waterman GF/F filter membrane and stored at −20 °C in dark. Chl *a* concentrations were measured by calibrated 10-AU-005-CE fluorometer (Turner Designs, USA) after the filters extracted with 90% acetone overnight at 4 °C (Parsons, Maita & Lalli, 1984). Samples for nutrient measurements, including DIN (NO_3_-N, NO_2_-N, NH_4_-N) and PO_4_-P, were collected in polypropylene bottles and analyzed with an AutoAnalyzer (BRAN and LUEBBE AA3, Germany).

### Statistic analysis

The data analyses were performed with SPSS 16.0 statistical program (SPSS Inc., Chicago, USA). After Saphiro–Wilk’s and Levene’s tests for normality and homogeneity, one-way ANOVA was used to examine the effects of the geographic locations on the abundance of the floating macroalgae and micro-propagules. Pearson correlation analyses were performed to investigate the correlations among abundance of floating algae and micro-propagules, and the environmental factors.

The redundancy analysis (RDA) was applied to assess the influence of environmental variables on the floating macroalgae and micro-propagules by CANOCO for Windows 5.0 (Šmilauer & Lepš, 2014).

## Results

### Spatial distribution and species composition of floating macroalgae

**Figure 2:**
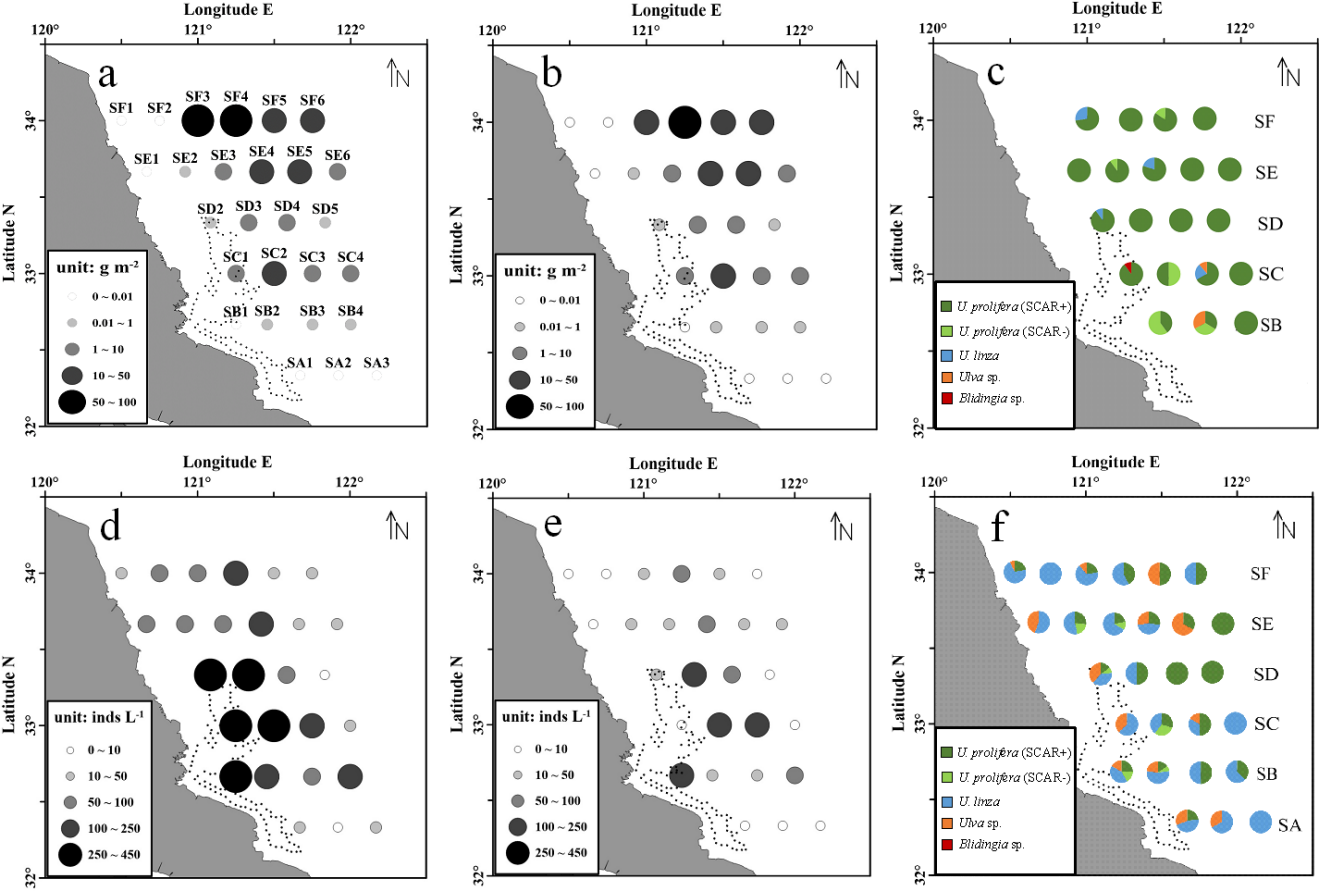
Abundance and species composition of the floating green macroalgae and micropropagules in Subei Shoal, southwestern Yellow Sea. Dotted line encloses the distribution of *Pyropia* aquaculture rafts. (A) The abundance of floating *Ulva* at each station; (B) the abundance of the ‘floating type’ of *U. prolifera*; (C) species composition of the floating green macroalgae; (D) the abundance of green macroalgal micro-propagules; (E) the abundance of micro-propagules of the ‘floating type’ *U. prolifera*; (F) species composition of the micro-propagules. Dotted line encloses the distribution of *Pyropia* aquaculture rafts.

During the survey period, the floating green macroalgae was widespread in the northern area of Subei Shoal (north to 32.5°N, Fig. 2A), and showed an evident spatial variation. Among the total 28 stations, floating *Ulva* was present at about 74% stations. No floating macroalgae was detected for the three stations at the south edge (SA1, SA2 and SA3) and those at the northwestern corner (SE1, SF1 and SF2). The average biomass of the floating macroalgae across all the stations was 12.8 g m^−2^. The biomass generally increased from south to north (*F* = 79.570, *P* < 0.01, one-way ANOVA), and the highest biomass was at the offshore stations of SF transect (34°N), off the Sheyang River Estuary.

According to the molecular analysis, there were four common green macroalgal species identified among the floating macroalgae, including *U. prolifera*, *U. linza*, *Ulva* sp. and *Blidingia* sp. *U. prolifera* was the dominant species throughout the survey region (Fig. 2C). The proportions of *U. prolifera* in the floating macroalgae ranged between 66.7% and 100% and were averaged 91.2%. The other species, *U. linza*, *Ulva* sp. and *Blidingia* sp., were relatively rare (4.7%, 3.5% and 0.6% in average, respectively). The specimen of *U. prolifera* was further differentiated into two ecotypes, the floating (SCAR+) and the normal (SCAR+) based on the specific SCAR marker (Zhao et al., 2015). The biomass of *U. prolifera* comprised 88.5% of ‘floating’ ecotype (SCAR+). Similar to the distribution pattern of total green macroalgae, ‘floating type’ of *U. prolifera* was most abundant at stations SF3–SF6 and SE4–SE5, in the northern survey region, and SC2 (Fig. 2).

### Spatial distribution and species composition of the micro-propagules

The green macroalgal micro-propagules were present throughout the survey area, and the abundance ranged from 6 to 432 inds L^−1^ (150 inds L^−1^ in average). The highest density was detected in the central region of Subei Shoal (SB1, SC1–SC2, SD1–SD2, >250 inds L^−1^, Fig. 2D), close to the *Pyropia* aquaculture rafts. And the abundance decreased rapidly from inshore to offshore (*F* = 73.270, *P* < 0.01, one-way ANOVA).

There were three *Ulva* species identified, *U. prolifera* (SCAR+ and SCAR −), *U. linza* and *Ulva* sp. Among the 870 micro-propagule seedlings identified into species level, 48.5% (in average) was *U. linza*, 35.1% was *U. prolifera* and 16.4% was *Ulva* sp. Approximately 88.0% of the *U. prolifera* propagules were ‘floating’ ecotype (SCAR+). Similarly, the micro-propagules of ‘floating type’ *U. prolifera* were abundant at the central Shoal, close to raft region (SB1, SC1–SC2, SD1, >250 inds L^−1^, Fig. 2E).

### The variations of SST, salinity, DO, Chl-a and nutrients

**Figure 3:**
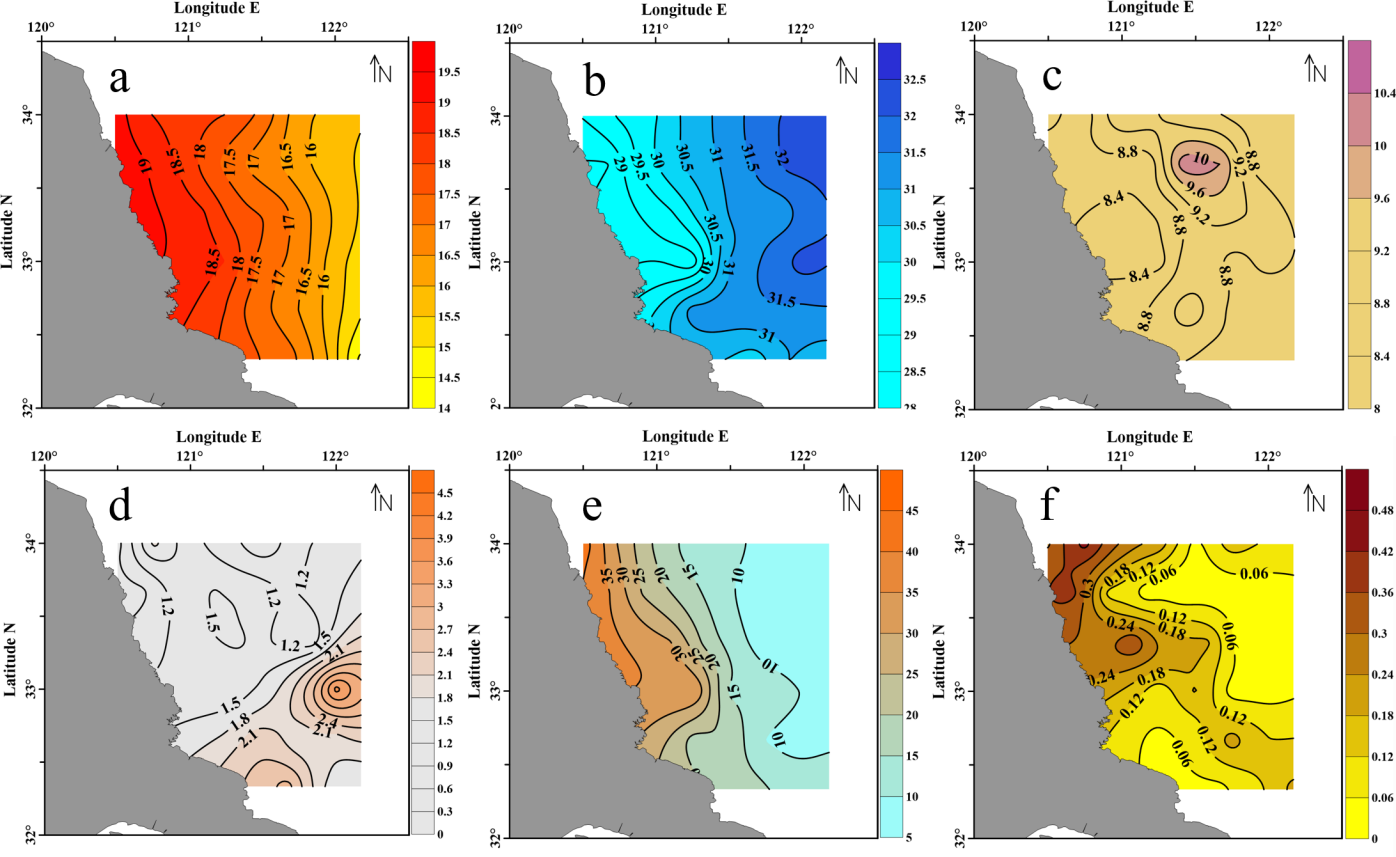
Surface seawater of the Subei Shaol, southwestern Yellow Sea. (A) SST; (B) salinity; (C) DO; (D) Chl *a*; (E) DIN and (F) PO_4_-P.

In the survey area, the SST varied from 14.4 °C to 19.2 °C, and was 17.2 °C in average. As shown in Fig. 3A, the SST obviously decreased from the inshore to offshore. In contrast, the values of salinity were generally low in the nearshore and high at the offshore (Fig. 3B), ranged from 28.5 to 32.2 psu (mean = 30.7 psu).

The dissolved oxygen (DO) in this region was 8.2–10.3 mg L^−1^, and 8.9 mg L^−1^ in average. The highest DO was observed at the northeastern corner of the survey area (Fig. 3C). The DO was converted to saturation (%) (C’_O2_, corrects for temperature and salinity-dependent oxygen solubility) using the Weiss equation (Riley & Skirrow, 1975). The results showed that DO was saturated throughout the sampled stations (C’_O2_ = 144–169%). The chlorophyll *a* (Chl *a*) concentrations varied from 0.7 µg L^−1^ to 3.7 µg L^−1^ with a mean of 1.6 µg L^−1^. The highest Chl *a* was detected at the southeast edge of the survey region.

The DIN concentrations ranged from 5.53–42.37 µmol L^−1^ with a mean of 17.20 µmol L^−1^. As indicated in Fig. 3E, the DIN concentrations along the coastline (>30 µmol L^−1^) were about 3 times higher than those in the eastern offshore water (<10 µmol L^−1^). And the decreasing trend of DIN towards the sea was consistently reverse to the salinity variation (Fig. 3B vs. 3e). The PO_4_-P concentrations varied from 0.02 to 0.44 µmol L^−1^ with a mean of 0.14 µmol L^−1^. Unlike DIN, the variation of PO_4_-P was patchy. The highest concentration was observed along the northern coast of the Shoal, close to the Sheyang River Estuary.

### Relationships among floating macroalgae, micro-propagules and the environmental parameters

Results of the correlation analyses among the floating algae and micro-propagules, and various environmental factors are summarized in Table 1. No evident correlation was detected between the biomass of floating algae (including the total green macroalgae and ‘floating type’ *U. prolifera*) and the micro-propagules (*r* = 0.036–0.086, *p* = 0.669–0.858). As described above, the floating *Ulva* macroalgae was prone to aggregate in the northern area, while high concentration of micro-propagules was found around the raft region. Statistic analysis further revealed a significantly negative correlation between the quantity of micro-propagules and the distances to the *Pyropia* aquaculture rafts (*r* = 0.469–0.656, *p* = 0.000–0.038), suggesting the influence of the rafts on the abundance of micro-propagules. The density of floating algae was positively associated with DO (*r* = 0.469–0.500, *p* = 0.008–0.014), and no other significant correlations were observed between the floating algae, micro-propagules and the environmental parameters (Table 1). At the same time, species compositions for floating macroalgae and micro-propagules were also distinct on both dominant species and spatial variation.

**Table 1:** Regression analyses (*r*, *p*) among the abundance of floating biomass and micro-propagules, and the environmental factors.

	Micro- propagule	Micro- propagule (SCAR+)	SST	Salinity	DO	Chl *a*	DIN	PO_4_-P	Distance
Floating biomass	0.036, 0.858		0.067, 0.738	0.155, 0.440	0.500, 0.008^**^	−0.328, 0.095	−0.271, 0.171	−0.011, 0.956	0.364, 0.062
Floating biomass (SCAR+)		0.086, 0.669	0.055, 0.787	0.165, 0.411	0.469, 0.014^*^	−0.349, 0.074	−0.284, 0.152	−0.28, 0.890	0.372, 0.056
Micro-propagule			0.311, 0.114	−0.152, 0.449	−0.244, 0.219	0.078, 0.697	0.221, 0.267	0.008, 0.968	−0.656, 0.000^**^
Micro-propagule (SCAR+)			0.068, 0.735	0.233, 0.243	0.011, 0.955	0.068, 0.738	−0.186, 0.352	−0.051, 0.802	−0.401, 0.038^*^
SST				−0.775, 0.000^**^	−0.105, 0.601	−0.355, 0.069	0.790, 0.000^**^	0.495, 0.009^**^	−0.194, 0.331
Salinity					0.237, 0.233	0.368, 0.059	−0.911, 0.000^**^	−0.604, 0.001^**^	0.240, 0.228
DO						−0.082, 0.686	−0.275, 0.166	−0.053, 0.794	0.236, 0.235
Chl *a*							−0.225, 0.259	−0.267, 0.178	−0.336, 0.086
DIN								0.567, 0.002^**^	−0.220, 0.270
PO_4_-P									0.101, 0.615

**Notes.**

Bold fonts indicate significant values of Pearson correlation.

* *p* < 0.05.

** *p* < 0.01.

Among the various environmental parameters, SST, salinity and nutrient concentrations (DIN, PO_4_-P) were closely correlated with each other (Table 1). As described above, there was a negative relationship between salinity and SST, while DIN and PO_4_-P concentrations were positively related with SST, indicating an obvious terrestrial input with fresh water.

**Figure 4:**
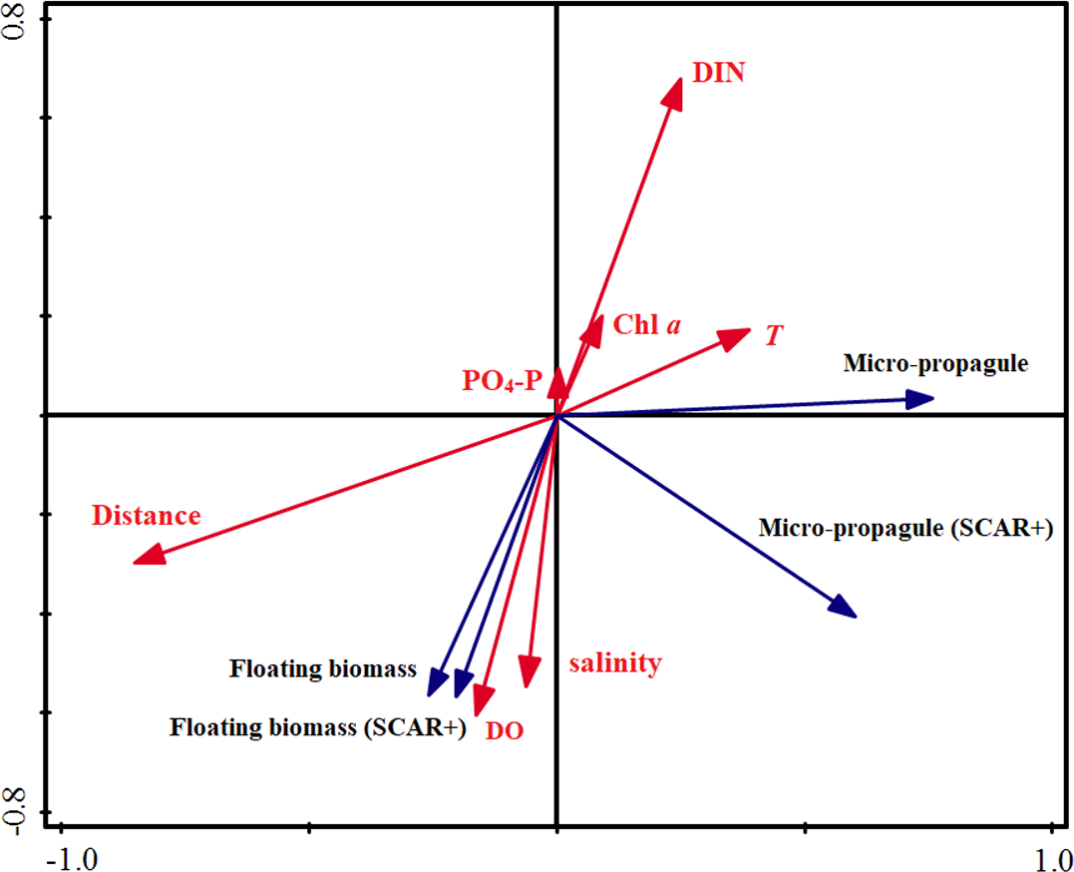
Redundancy analysis (RDA) on the abundance of floating Ulva macroalgae, micropropagules and the environmental variables.

Similar to the correlation analyses, the RDA results indicated that distance to the rafts was the most important factor influencing the abundance of the micro-propagules, and explained 43.0% of the variance in total (Table 2). The DO was closely associated with the distribution of the floating algae and explained 33.0% of the total variance. The remaining factors explained only 15.4% (micro-propagules) and 20.3% (floating algae) of the total variance, respectively. Based on the perpendicular projection and angle, the Fig. 4 confirmed that abundance of micro-propagules was negatively associated with the distances, and the biomass of floating algae was positively correlated with DO.

**Table 2:** Variation partitioning analysis of the environmental factors on the green macroalgae in Subei Shoal of Yellow Sea in 2016.

Environmental factor	Eigenvalues	Variation explains solely /%	*F*	*p*
Micro-propagule				
SST	0.035	3.5	1.6	0.226
Salinity	0.052	5.2	2.5	0.108
DO	0.004	0.4	0.2	0.770
Chl *a*	0.024	2.4	1.1	0.320
DIN	0.029	2.9	1.4	0.280
PO_4_-P	0.010	1.0	0.5	0.442
Distance	0.430	43.0	18.9	0.002
Floating biomass				
SST	0.079	7.9	3.4	0.082
Salinity	0.017	1.7	0.7	0.410
DO	0.330	33.0	12.3	0.004
Chl *a*	0.066	5.4	2.6	0.108
DIN	0.019	1.9	0.7	0.428
PO_4_-P	0.005	0.4	0.3	0.656
Distance	0.030	3.0	1.2	0.200

## Discussion

To study the early development process of green-tides in Yellow Sea and the associated influencing factors, we surveyed the spatial distribution of floating green macroalgal biomass and amount of micro-propagules in Subei Shoal and further analyzed the relationships among the environmental and biological parameters. The results illustrated distinct distributional patterns of floating green macroalgae and micro-propagules. The former was abundant in the northern region of the Subei Shoal, while the latter was often accumulated around the central raft region. The most important factor influencing the abundance of micro-propagules was the distance to the rafts, while the biomass of floating algae was found closely related to DO. The significance and underlying reasons for the distributional pattern and environmental variations were discussed as following.

The distribution of biomass of floating algae indicated the integrated influences of the source and environmental factors on this parameter. The biomass at 6 stations was relatively high (>30 g m^−2^), among which five were located in the north (SE and SF transects) and one (SC2) was close to the east edge of the raft region. Considering the consistent biomass source from the aquaculture rafts (Liu et al., 2009; Liu et al., 2010; Liu et al., 2013; Wang et al., 2015; Zhang et al., 2017), the high biomass at SC2 was due to the green macroalgal wastes discarded from rafts. Whereas, the survey period in this research (May 15th–20th) was almost at the end of the raft-cleaning and recycling (Xing et al., 2019). The limited green macroalgal source in this period could result in generally low biomass at the stations around the raft region. Subsequently, the algae were drifted northwardly driven by the monsoonal wind and surface current, and their biomass increased with time. (Bao et al., 2015). Consistent with the satellite remote sensing data from other years (Yuan, Xiao & Zhang, in press), high biomass was often found accumulating in the northern region around 34°N, outside the Sheyang River Estuary (Fig. 2A). This was probably resulted from the rapid biomass growth supported by high nutrient input with fresh water. Li et al. (2015) also noted that river discharge from Sheyang and Guanhe contributed substantial nutrients along the coasts of southwestern YS, and supported the green macroalgal bloom in this region. Furthermore, slightly low water flow in this northern open mouth may assist the pileup of the floating algae after it was flushed out through the shallow sand grooves (Bao et al., 2015). Wind direction and speed could greatly influence the distribution of the floating algae in the northern open water, out of Subei Shoal (Qi et al., 2016; Yuan, Xiao & Zhang, in press). At the same time, that no floating macroalgae were detected at the nearshore stations (SE1, SF1, SF2, Fig. 2A) indicated that the high biomass around 34°N was not derived from the terrestrial aquaculture ponds or the western coastline in this region (Pang et al., 2010), but from the intertidal shoal area at the southwest.

Different from the floating macroalgae, the micro-propagules showed high abundance in the central Shoal, around the aquaculture rafts area, suggesting significant impacts from the *Pyropia* aquaculture rafts. This result was congruent with the previous studies, which found year-round existing of the micro-propagules in the Subei Shoal and supported the function of aquaculture rafts on providing and maintaining the high quantity of micro-propagules in this region (Song et al., 2014; Liu et al., 2018; Miao et al., 2018; Miao et al., 2020). Besides, the obviously distinct species composition and distributional pattern of the micro-propagules, compared to the floating macroalgae, implied little direct contribution of micro-propagules for the biomass of floating algae in this region. Interestingly, the distribution and species composition of the micro-propagules varied significantly in different regions. A number of field surveys in western YS (including Subei Shoal, open water in western part of the south YS, nearshore coasts of Shandong, Jiangsu provinces etc.) revealed that distribution of the micro-propagules in open water were closely related to the floating algae and *U. prolifera* dominated (Li et al., 2014; Huo et al., 2016), while the micro-propagules in Subei Shoal and along the coasts comprised diverse species (*U. linza*) and showed evident seasonal fluctuations (Liu et al., 2018; Miao et al., 2018; Miao et al., 2020, this research). All these facts implied distinct origins of the micro-propagules in different regions and their interactions with the attached and floating algae. The rafts (or the attached green macroalgae) contributed substantial amount of micro-propagules to the water column, which overcame those from the floating macroalgae in Subei Shoal. The functions of the micro-propagules in the YSGT probably need to be investigated and discussed based on the regions and stages of the green tides.

In addition, a significant correlation between the biomass of floating algae and DO was observed, which suggested active photosynthesis of the initial floating macroalgae and the contribution for the dissolved oxygen in the Subei Shoal. Comparative studies found distinct morphology and physiological activities of the floating *U. prolifera* in the source region and at the late stage of the YSGT (Zhang et al., 2013). It is obvious that high chlorophyll contents and great physiological activity could lead to high photosynthesis, and then rapid growing and strong buoyancy ability of the initial floating algae in Subei Shoal (Zhang et al., 2013; Wang et al., 2015; Fu et al., 2019). The physiological advances (e.g., higher nutrient assimilation, growth, photosynthetic and buoyancy abilities etc.) of the species *U. prolifera* could further boost the blooming and dominance of this species in open waters (Fu et al., 2019; Fort et al., 2020; Liu et al., 2020)

No significant correlation was detected between the micro-propagule, biomass of floating algae and the other environmental factors, e.g., seawater temperature, nutrients. Series of laboratory experiments, remote sensing analyses, field surveys and numeric modeling suggested that both sea surface temperature and nutrients are critical for the green tide initiation. An overall surface temperature >15° C was consistently observed for the initiation of the green tides through the years (Wu, Xu & Wu, 2000; Zhang et al., 2009; Liu et al., 2010; Yuan, Xiao & Zhang, in press), and required for the rapid growth of *U. prolifera* thalli (Song et al., 2015; Xiao et al., 2016). In addition, the high nutrient level of the coastal water, especially the consistently increased nitrogen during the past 40 years, was the important contributor for the large-scale green tides in Yellow Sea (Li et al., 2015; Shi et al., 2015). Xiao et al. (2019) also found that terrestrial nutrient input was related to the overall scale of the green tide in certain years. Apparently, suitable seawater temperature and sufficient nutrients were prerequisites for the *U. prolifera* bloom. Laboratory testing and numerical modeling indicated that minimum concentrations for the growth of *U. prolifera* were 6.5 µmol L^−1^ for nitrate and 0.27 µmol L^−1^ for phosphate (Wang et al., 2019; Wang et al., 2020). The field data from the current and a number of other studies showed that both temperature (14.4–19.2 °C, mean = 17.2 °C) and nutrients (nitrate, 5.53–42.37 µmol L^−1^, mean = 17.20; phosphate, 0.02–0.44 µmol L^−1^, mean = 0.14) in Subei Shoal were higher than the above limiting line. Therefore, the seawater temperature and nutrients in the Subei Shoal were suitable for the bloom, and not the key factor limiting expansion and distribution of the floating macroalgae. Further extensive research covering a wider geographic range, including the regions with relatively low nutrients and cold water, would be able to analyze the function of temperature and nutrients on constraining the distribution and expansion of the green tides.

## Concluding Remarks

The small-scale field research revealed distinct spatial distributions and dominance patterns of the floating macroalgae and micro-propagules in the Subei Shoal where the large-scale green tide in Yellow Sea is initiated. The floating macroalgal biomass was abundant in the northern region of the shoal, which was probably driven by the local geochemical and physical environment, such as northward wind and sea surface circulation, nutrient level influenced by the river discharge etc. The micro-propagules, however, were mostly accumulated around the aquaculture rafts and decreased rapidly with the distance to the rafts, which suggest the importance of the aquaculture rafts on maintaining the propagule populations in the shoal. This distinct distribution and species composition of micro-propagules and floating macroalgae indicated little direct contribution from the environmental micro-propagules to the floating macroalgal biomass in the shoal. Consistent with previous studies, the micro-propagules selectively favor growing on the aquaculture facilities and undergo series of development processes before they could turn into floating macroalgae. These development processes are influenced by a number of biological and anthropogenic factors (e.g., rapid increase of temperature and light availability in spring, the raft-cleaning activities etc.). The ‘floating’ type of *U. prolifera* dominates the attached green macroalgal community and subsequently the floating macroalgae with its physiological advantages under this kind of environmental selection, such as high nutrient assimilation, photosynthesis and growth rate, strong buoyancy ability (Fu et al., 2019; Wang et al., 2019; Liu et al., 2020). It is worth noting that the abundance and dominance pattern of micro-propagules in Subei Shoal are quite different with those in open water, where the abundance and species composition of micro-propagules are closely associated with floating macroalgae (Li et al., 2014; Huo et al., 2014; Huo et al., 2016). This discrepancy suggested distinct sources or reservoirs of propagules and their relationships with floating macroalgae. Further research is needed to discuss or investigate the different functions of propagules on bloom forming in different regions. Continuous monitoring on the micro-propagule populations, the initial floating macroalgae and environmental conditions in Subei Shoal is also necessary for predicting or forecasting any variation of the green tides in YS in the long run.

##  Supplemental Information

10.7717/peerj.10538/supp-1 Supplemental Information 1 The raw measurements of the abundance of floating Ulva macroalgae, micropropagules and the environmental variables

10.7717/peerj.10538/supp-2 Supplemental Information 2 The raw measurements of the species compositions of floating macroalgae amd micro-propagules

## Additional Information and Declarations

### Data Availability

The following information was supplied regarding data availability: The raw measurements are available in the Supplementary Files.
